# Dietary Sialyllactose Does Not Influence Measures of Recognition Memory or Diurnal Activity in the Young Pig

**DOI:** 10.3390/nu10040395

**Published:** 2018-03-23

**Authors:** Stephen A. Fleming, Maciej Chichlowski, Brian M. Berg, Sharon M. Donovan, Ryan N. Dilger

**Affiliations:** 1 Piglet Nutrition and Cognition Laboratory, Department of Animal Sciences, University of Illinois, Urbana, IL 61801, USA; sflemin2@illinois.edu; 2 Department of Animal Sciences, University of Illinois, Urbana, IL 61801, USA; 3 Mead Johnson Pediatric Nutrition Institute, Evansville, IL 61142, USA; maciej.chichlowski@rb.com (M.C.); dr.brianberg@gmail.com (B.M.B.); 4 Division of Nutritional Sciences, University of Illinois, Urbana, IL 61801, USA; sdonovan@illinois.edu; 5 Department of Food Science and Human Nutrition, University of Illinois Urbana-Champaign, Urbana, IL 61801, USA

**Keywords:** pig, nutrition, brain, development, sialyllactose, sialic acid, oligosaccharide, cognition

## Abstract

Sialic acid (SA) is an integral component of gangliosides and signaling molecules in the brain and its dietary intake may support cognitive development. We previously reported that feeding sialyllactose, a milk oligosaccharide that contains SA, alters SA content and diffusivity in the pig brain. The present research sought to expand upon such results and describe the effects of feeding sialyllactose on recognition memory and sleep/wake activity using a translational pig model. Pigs were provided ad libitum access to a customized milk replacer containing 0 g/L or 380 g/L of sialyllactose from postnatal day (PND) 2–22. Beginning on PND 15, pigs were fitted with accelerometers to track home-cage activity and testing on the novel object recognition task began at PND 17. There were no significant effects of diet on average daily body weight gain, average daily milk intake, or the gain-to-feed ratio during the study (all *p* ≥ 0.11). Pigs on both diets were able to display recognition memory on the novel object recognition task (*p* < 0.01), but performance and exploratory behavior did not differ between groups (all *p* ≥ 0.11). Total activity and percent time spent sleeping were equivalent between groups during both day and night cycles (all *p* ≥ 0.56). Dietary sialyllactose did not alter growth performance of young pigs, and there was no evidence that providing SA via sialyllactose benefits the development of recognition memory or gross sleep-related behaviors.

## 1. Introduction

Human milk has been shown to have numerous benefits in comparison to infant formula in stimulating the growth and development of gastrointestinal and immune systems [1]. A recent meta-analysis suggests that breastfeeding promotes cognitive development [2], but the mechanisms and strength of the relationship are unclear [3]. There is mounting evidence that components of human milk such as DHA [4], choline [5], and gangliosides [6] support brain development, and emerging research suggests milk oligosaccharides (ranging from 3–32 monosaccharide units in length [7]) may contribute to brain development as well [8]. Some milk oligosaccharides may act as prebiotics [8], and we recently demonstrated that pigs fed a combination of prebiotics demonstrated increased exploratory behavior and improved recognition memory [9]. Milk oligosaccharides are a heterogeneous group of oligosaccharides with diverse functions largely related to immunity and gut physiology [8]. The composition of human milk oligosaccharides is 10–20% sialylated [8]. As sialic acid (SA) is present at relatively high concentrations in the brain as a part of gangliosides and signaling molecules that regulate neurodevelopment [6], the impact of sialylated oligosaccharides such as sialyllactose (SL) is of interest as they may support brain development.

Supplementation with SA-containing ingredients, including complex milk lipids [10], gangliosides [11], casein glycomacropeptide (cGMP) [12], lactoferrin [13], and SL [14], has been shown to improve cognition or alter stress-related behaviors. In a young adult mouse model investigating possible anxiolytic effects of SL, both 3′ and 6′ isomers reduced anxiety-related measures and restored performance to control levels when mice were introduced to a social stressor. Furthermore, SL attenuated stress-related sleep disruptions in adult rats [15] and tended to increase performance on spatially-based behavioral tasks [16]. A recent study demonstrated that pigs fed SL-supplemented formula for 21 days had greater total SA concentrations in the corpus callosum when fed milk containing 2 g/L of either 3′- or 6′-SL compared with pigs provided formula containing no SL or 4 g SL/L milk [17]. Similarly, a previous study from our lab showed that of a range of doses of SL, ranging from 55–779 mg SL/L of formula, a moderate dosage of 429 mg SL/L increased free-to-bound hippocampal SA, reduced bound SA in the prefrontal cortex, and increased mean, axial, and radial diffusivity in the corpus callosum [18]. Taken together with the findings of Jacobi and colleagues [17], these results show that feeding SL results in dose-dependent, structural, and region-specific increases in brain SA, but it remains to be shown whether this has functional consequences for behavior. Accordingly, our hypothesis in the present study was that supplementation with a moderate dosage of 380 mg SL/L would improve the performance of pigs on the novel object recognition task and influence measures of sleep/wake activity. We chose to supplement the diet at 380 mg SL/L, which is within the concentration range found in mature human milk [19].

## 2. Materials and Methods

### 2.1. Animals and Housing

All animal care and experimental procedures were in accordance with the National Research Council Guide for the Care and Use of Laboratory Animals and approved by the University of Illinois at Urbana-Champaign Institutional Animal Care and Use Committee. Approval for this research project was verified on 3 March 2015 and is identified as IACUC 15034 at the University of Illinois Urbana-Champaign. Beginning on postnatal day (PND) 2, naturally-farrowed, intact male pigs (*n* = 36) were artificially-reared through PND 22. The trial was completed in one replicate with 18 pigs per diet, selected from 9 L to control for genetics (same sire and related dams between litters) and initial bodyweight. All pigs were provided a single subcutaneous 5 mL dose of *Clostridium perfringens* antitoxin C and D (Colorado Serum Company, Denver, CO, USA) on PND 2. If health status appeared compromised (i.e., diarrhea, lethargy, elevated body temperature), an additional 5 mL dose of *C. perfringens* antitoxin C and D was administered orally until symptoms resolved; a total of seven pigs were given additional doses of antitoxin. Housing, temperature, and lighting were conducted as described previously [18]. Two pigs were euthanized prior to the conclusion of the study due to insufficient weight gain and failure-to-thrive (*n* = 1/diet). Data from the remaining 34 pigs (*n* = 17/diet) were used for subsequent analyses and are presented herein.

### 2.2. Dietary Groups

All diets were produced by Mead Johnson Nutrition (Evansville, IN, USA) using a proprietary blend of nutrients formulated to meet the nutritional needs of growing pigs. Pigs were provided one of two custom diets from PND 2–22. The control diet (Control) included docosahexaenoic acid (DHA, 87 mg/100 g milk replacer powder; DSM, Heerlen, The Netherlands), arachidonic acid (ARA, 174 mg/100 g milk replacer powder; DSM, Heerlen, The Netherlands), galactooligosaccharide (GOS, 1.0 g/100 g milk replacer powder; FrieslandCampina, Zwolle, The Netherlands), and polydextrose (PDX, 1.0 g/100 g milk replacer powder; Danisco, Terre Haute, IN, USA). The experimental diet (Sialyllactose) was formulated using the Control diet as the base and supplemented with bovine-derived modified whey enriched with SL (SAL-10; Arla Foods Ingredients, Aarhus, Denmark) to provide a final SL concentration of 190 mg SL/100 g milk replacer powder.

Milk replacer powder was reconstituted fresh each day at 200 g of dry powder per 800 g of water. At this reconstitution rate, all diets provided equal concentrations of DHA (174 mg/L), ARA (348 mg/L), and PDX/GOS (each at 2 g/L). The reconstituted milk replacers were formulated to contain 0 mg SL/L (Control) or 380 mg SL/L (Sialyllactose). Pigs were fed ad libitum using an automated milk replacer delivery system that dispensed milk from 10:00 to 06:00 the next day. Leftover milk from the previous day and individual pig bodyweights were recorded daily. The remaining volume of milk was subtracted from the initial volume provided to quantify milk disappearance following the 20-h feeding period, which will henceforth be referred to as milk intake. Milk intake from PND 21 was omitted from analyses as pigs were fasted overnight prior to the end of study on PND 22. An electrolyte solution (Swine Bluelite, Tech Mix, Stewart, MN, USA) was provided to all pigs from PND 2–5 to help maintain electrolyte balance and avoid dehydration.

### 2.3. Behavioral Testing

#### 2.3.1. Novel Object Recognition

The novel object recognition (NOR) task was used to assess object recognition memory. Testing consisted of a habituation phase, a sample phase, and a test phase. During the habituation phase, each pig was placed in an empty testing arena for 10 min each day for two days leading up to the sample phase. In the sample phase, the pig was placed in the arena containing two identical objects and given 5 min for exploration. After a delay of 48 h, the pig was returned to the arena for the test phase. During the test phase, the pig was placed in the arena containing one object from the sample phase as well as a novel object and allowed to explore for 5 min. Between trials, objects were removed, immersed in hot water with detergent and rubbed with a towel to mitigate odor, and the arena was sprayed with water to remove urine and feces. Objects chosen had a range of characteristics (i.e., color, texture, shape, and size); however, the novel and sample objects only differed in shape and size. Only objects previously shown to elicit a null preference were used for testing. Habituation trials began at PND 17 and testing on the NOR task began at PND 19. The amount of time exploring objects and distance moved was measured using a combination of automated procedures using Ethovision (Ethovision XT 11^®^, Noldus Information Technology, Wageningen, The Netherlands) and manual tracking (for a review of each measure assessed, see Fleming and Dilger [20]). The recognition index, the proportion of time spent with the novel object compared to total exploration of both objects, was used to measure recognition memory. A recognition index significantly above 0.50 demonstrates a novelty preference and, thus, recognition memory. Trials were removed from analyses if experimental/technical errors existed or pigs explored either object for less than 2 s during the sample or test trial. If pigs did not explore either object for greater than 2 s during the sample trial, they were also removed from analysis during the test trial regardless of performance. Two and five pigs provided the Control and SL diets, respectively, did not meet the above criteria and were removed from the final analysis (final *n* = 15, Control; *n* = 12, Sialyllactose).

#### 2.3.2. Activity Analysis

Accelerometers (Actiwatch 2, Philips Respironics, Bend, OR, USA) were secured to collars and fastened around each pig’s neck between PND 15 and 22 (*n* = 12 per diet) and were set to sample movement every 15 s. Only periods where full day and night cycles were recorded were used for analysis (PND 15 and 22 were omitted as collars were only on for part of the day, for a remaining total of six full cycles between PND 16 and 21). When pigs were found without collars, the collar was re-applied and the time was noted. After study completion, home-cage video was used to verify that periods of complete inactivity were due to the loss of the collar, and these times were also removed from analysis. For the analysis of sleep/wake outcomes, specialized software (Actiware 6.0.7, Philips Respironics, Murrysville, PA, USA) was used to calculate a unique wake threshold value (used to determine if the pig was asleep or awake based on movement during 2-min periods before and after a single 15-s epoch) for each pig and quantify the total activity count and percent time asleep. Data were collected for six consecutive days and sleep outcomes were assessed as averages across that period.

A preliminary analysis was conducted to assess the validity of sleep scores in pigs from actigraphy data. Approximately one hour of activity data collected from six pigs was scored by actigraphy software as compared to the manual scoring of recorded video. Video was split into 15-s epochs, for a total of 247 epochs, and manually analyzed. If a pig was visually-assessed as asleep for more than 50% of a single 15-s epoch, that epoch was classified as a “sleep” epoch. Epochs were chosen by selecting for periods of apparent transition between sleep and wakefulness as these are the most difficult to classify and appeared to be most variable between manual and automatic scoring methods.

### 2.4. Statistical Analysis

All data generated as part of this study were subjected to an analysis of variance (ANOVA) using the MIXED procedure of SAS Enterprise Guide 5.1 (SAS Institute Inc., Cary, NC, USA). Depending on the outcome, one of two statistical models was used: (1) data collected at a single time-point (e.g., average body weight gain over the entire study, performance in the NOR test trial) were analyzed by one-way ANOVA; and (2) data collected from the same animal on more than one occasion (e.g., diurnal activity) were analyzed using two-way repeated-measures ANOVA. Litter was included as a random effect in both statistical models. For NOR testing, a one-tailed *t*-test was conducted to assess if recognition index was greater than 0.5 (i.e., random chance). In all instances statistical significance was considered at *p* < 0.05.

## 3. Results

### 3.1. Diet Composition

Analytical assessment conducted after study completion showed levels of SL in the experimental diets were close to formulated levels (374 mg SL/L vs. 380 mg SL/L in the Sialyllactose diet). However, the Control diet contained 58 mg SL/L due to endogenous SL in the bovine milk ingredients. Energy, macronutrient, and micronutrient composition were comparable between Control and Sialyllactose diets (see Table 1 for analyzed nutrient composition).

### 3.2. Growth Performance and Health Status

**Figure 1:**
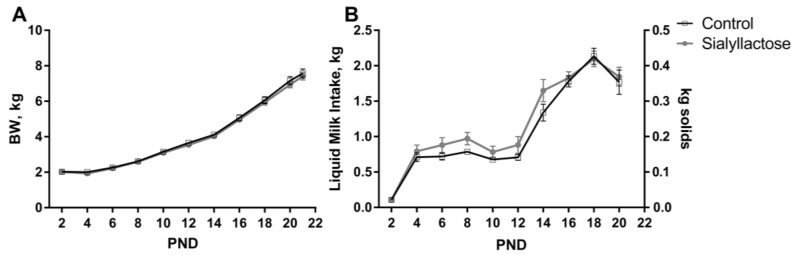
Body weight (BW) (**A**) and liquid milk intake (**B**) during the trial. No differences in average daily body weight gain, average daily milk intake, or the feed efficiency ratio (i.e., body weight gain:feed intake ratio) were observed between groups (*p* ≥ 0.11). Data for milk intake on postnatal day (PND) 22 are not shown as piglets were fasted overnight prior to the end of study.

No differences were observed for average daily body weight gain, average daily milk intake, or the feed efficiency ratio (i.e., gain-to-milk intake) between diets across the duration of the study (all *p* ≥ 0.11, Table 2, Figure 1). Additionally, daily health checks revealed low incidence of loose stool in pigs and no differences in pig health status or compliance to consume experimental dietary treatments. Thus, all pigs remained healthy throughout the study duration and both dietary treatments were equally well tolerated by pigs as evident in the observed trajectory of body weight gain.

### 3.3. Novel Object Recognition

Regardless of dietary treatment, all pigs were able to display recognition memory in the NOR test trial (*p* < 0.01, Table 3). However, there were no differences between dietary treatment groups for measures of exploratory behavior, most notably time spent investigating objects, number of object visits, and mean time spent per object visit (all *p* ≥ 0.11, Table 4). Although some pigs were removed due to non-compliance, ultimately our study was powered to capture an effect size of 0.89 with a power of 0.80 when evaluating differences in the NOR recognition index.

### 3.4. Activity Analysis

Validation of the automated scoring method (i.e., computer-assisted analysis of actigraphy data) against the manual scoring of home-cage video was performed. A chi-square test for equality of two proportions showed that automated and manual scoring methods were not different (*p* = 0.065), with the automated scoring being only 7% more likely to score an epoch as “sleep” and less likely to score an epoch as “awake”. Therefore, with the validation of the automated actigraphy data scoring method complete, all sleep/wake activity reported herein was generated using the automated software-based method. In general, there was no significant main effect of diet or interaction effect of diet by cycle for total activity or percent time asleep (all *p* ≥ 0.56, Table 5). While intuitive, total activity counts and time asleep were both influenced by cycle (i.e., day vs. night; both *p* < 0.01).

## 4. Discussion

Siallylactose is one of several sources of SA, and comparisons of mature human and porcine milk demonstrate that the SA content of human milk is much greater than that of porcine milk. Mature human milk provides approximately 500 mg SA/L milk [21], porcine milk contains approximately 10 mg SA/L milk [22], and infant formula falls between 65–290 mg SA/L milk [23] (for a thorough review of SA content of milk and other food products, see Röhrig et al. [24]). In this study, a dose of 380 mg SL/L was tested as a previous study reported that this dose was most effective at eliciting changes in SA content and diffusivity in the brain [18]. This dose is well below the SL dose that was shown by Jacobi et al. [17] to enrich corpus callosum and hippocampal SA content [17], but is within the range found in mature human milk [19]. Here, the impact of dietary SL on growth, recognition memory, exploratory behavior, and diurnal activity was investigated, but no impact of diet was observed for any measure.

Pigs fed an SL-supplemented diet did not display altered sleep behavior, whereas a past report demonstrated that SL attenuated disruptions to sleep architecture after exposure to an acute inescapable stress [15]. An important distinction from past research is that diurnal activity was measured in a minimally stressful environment, whereas SL may provide a neuroprotective effect that is only observed in the context of a more extreme stressor. Additionally, Chichlowski et al. [15] used electroencephalography (EEG), allowing the experimenters to more accurately quantify the timing, stage, and quality of sleep. Although both treatment groups in our study had similar activity during the day and night, differences in the quality of sleep may have been observed if assessments were made using EEG.

While different doses and/or longer duration of SL administration may have produced a cognitive benefit, it is possible that supplemental SA (via SL) was not required in the behavioral paradigm under which the pigs were assessed. Active learning increases the expression of mRNA for the enzyme critical for regulating SA biosynthesis (UDP-N-acetylglucoasamine-2-epimerase/*N*-acetylmannosamine kinase, GNE) by 2- to 3-fold in the hippocampus and liver of pigs [25]. As the NOR task makes use of spontaneous behavior rather than operant conditioning the cognitive load required to learn a rule was not present, and there may not have been a physiological demand for increased SA utilization in the brain. Our data show that dietary SL supplementation did not provide a cognitive benefit as assessed by NOR, which conflicts with previous work that showed young pigs exhibit cognitive benefits from dietary SA supplementation when using behavioral tasks dependent on operant conditioning [12,13]. Moreover, these results may reflect the presence of prebiotics in both the control and experimental diet. We previously reported that pigs fed milk replacers containing the prebiotics PDX and GOS demonstrated increased exploratory behavior and improved recognition memory using the same behavioral paradigm (i.e., the NOR task with a delay of 48 h) [9]. Together with evidence that piglets are capable of performing the NOR task at younger ages using shorter delays [20], we believe the difficulty of the task was appropriate. Rather, the potential cognitive benefits from SL may have been masked by the inclusion of PDX and GOS in each diet.

To our knowledge, few studies have evaluated the ability of dietary SL to affect behavior and cognition. Tarr and colleagues [14] found that dietary SL attenuated stressor induced anxiety-like behaviors in rats and preliminary data suggests that SL may prevent stress-induced alterations in sleep architecture [15]. However, another report in rats demonstrated that feeding SL only produced a non-significant trend towards improved cognition on spatial tasks [16]. The majority of data suggesting dietary SL may be beneficial for cognitive development come indirectly from studies that investigated other SA-containing ingredients such as gangliosides [10,11,26], cGMP [12], and lactoferrin [13]. Each of these ingredients vary in structure and function, but are common in that they contain SA. Gangliosides are sialylated glycosphingolipids highly concentrated in the brain [6], cGMP is an SA-enriched peptide released during the formation of cheese from the protein kappa-casein [27], and lactoferrin is an iron binding glycoprotein enriched in SA with various functions related to iron metabolism [28].

Gangliosides contribute 75% of conjugated SA in the brain where they play a critical role in functions such as synaptic transmission, plasticity, neurogenesis, synaptogenesis, cell proliferation, and cell differentiation [6]. Exogenous, but not dietary, gangliosides and SA appear to be effective at promoting cognition in adult or aging models [29,30,31,32,33] and deficit (i.e., drug-induced amnesia, cortical lesions, malnourishment) models [34,35,36]. The impact on young, normal animals is mixed, but ganglioside and SA administration have shown both positive and neutral effects on cognition [31,37]. These studies provided preliminary evidence that exogenous SA improves cognition; however, there is less evidence that dietary gangliosides improve cognition in normative models (i.e., gangliosides are provided at physiological concentrations via the diet to healthy animals during typical development).

Male and female pigs fed formula containing a mix of 0.8% or 2.5% phospholipids and gangliosides displayed fewer errors in a spatial T-maze test compared with controls and had larger brain weights. Furthermore, volumes of several brain regions including the internal capsule, putamen, and thalamus appeared sensitive to supplementation [11]. As has been discussed for SA, the cognitive effects of dietary ganglioside supplementation appear dependent on dosage and behavioral task employed. Rats provided complex milk lipid in doses of 1.0% but not 0.2% exhibited greater behavioral performance in the novel object recognition and Morris water maze, but no improvement in operant conditioning tasks [10]. A subsequent report from the same group demonstrated that even lower doses of 0.05% and 0.01% had no effect on operant learning, and spatial or recognition memory [38]. In a separate study, dietary gangliosides fed to children with cerebral palsy for 3 months elicited improved muscle tension, limb function, language ability, and intelligence [26]. Due to the use of a developmentally-appropriate preclinical model for the human infant, this study provides strong evidence that gangliosides contribute to not only cognition but also motor function and development. Taken together these data suggest that, provided the correct dosage, gangliosides fed alone or as part of a complex milk lipid may have the capacity to promote cognitive development.

Young pigs supplemented with SA via cGMP from PND 3–35 displayed dose-dependent increases in performance in the radial arm maze, with those provided the most cGMP completing the difficult version of the task with the fewest mistakes. All groups fed cGMP had enriched protein-bound, but not ganglioside-bound, SA in the frontal cortex. Additionally, pigs fed the highest amount of cGMP had increased levels of *ST8SIAIV*, a polysialyltransferase important in SA metabolism. After a correlational analysis, it was revealed that sialyltransferase activity in the frontal cortex correlated inversely with number of mistakes on the behavioral task, with pigs exhibiting lower sialyltransferase activity making more mistakes. Despite this correlation, there were no dietary effects on sialyltransferase activity, suggesting sialyltransferase activity may not be involved in the mechanism by which performance was improved. In a later study by the same group, pigs supplemented with lactoferrin from PND 3–38 were found to have increased performance on the radial maze. More pigs in the treatment group were able to complete both easy and difficult versions of the task, with the pigs provided lactoferrin making fewer mistakes on the difficult version [13]. Gene microarray data in hippocampal tissue revealed that pigs fed lactoferrin had upregulated expression of genes in the brain-derived neurotrophic factor (BDNF) neurotrophic signaling pathway, affecting genes related to organization of the cytoplasm, cytoskeleton, growth of neurites, and anxiety [13]. The finding that the provision of dietary lactoferrin influenced the expression of genes related to anxiety suggested that this protein may decrease anxiety, which aligns with the previously discussed results from Tarr et al. [14], wherein mice provided SL demonstrated attenuated anxiety when introduced to a social stressor.

Overall, few studies have evaluated the impact of SL on cognition and behavior. However, there is evidence that SA-containing ingredients positively influence cognitive performance, but making cross-sectional comparisons is confounded by the use of several different SA-enriched ingredients. While containing SA, SL, gangliosides, cGMP, and lactoferrin differ vastly in structure and function, contributing to the variation between study results.

Although our intervention coincided with a significant portion of brain growth in the pig, our investigation may have been limited by the duration of the trial, which may not have allowed sufficient time for SL to confer cognitive benefits. As discussed, the novel object recognition task may not reflect a context wherein supplemental SA is beneficial, and comparisons between spontaneous and operant behavior may elucidate the conditions that lead to increased neural requirements for SA. While clinically translatable, the use of actigraphy instead of EEG did not allow the examination of neural activity during sleep and the quantification of sleep stages, thus our measures were only a gross representation of sleep activity. Lastly, although the goal of this study was to evaluate the efficacy of 380 mg SL/L at supporting cognitive development, we cannot exclude the possibility that a higher supplementation dose or longer supplementation period may have elicited cognitive benefits.

## 5. Conclusions

While there are several reports that SA-containing ingredients may influence cognitive development, we found no evidence that bovine-derived dietary SL provided at 380 mg SL/L was effective at altering recognition memory or sleep-related activity.

## Figures and Tables

**Table 1:** Analyzed nutrient composition of experimental diets.

Nutrient per Liter	Control	Sialyllactose
Sialyllactose, mg	58	374
Energy and macronutrients		
Total calories, kcal	1049	1020
Carbohydrate, g	57	58
Fat, g	64	60
Protein, g	61	62
Minerals		
Calcium, mg	2233	2178
Chlorine, mg	1141	1158
Copper, μg	1640	1505
Iodine, μg	274	271
Iron, mg	19	19
Magnesium, mg	227	241
Manganese, μg	2305	2159
Phosphorus, mg	1621	1673
Potassium, mg	2255	2349
Selenium, μg	65	68
Sodium, mg	1708	1708
Zinc, mg	17	17
Vitamins and other nutrients		
Vitamin A, IU	4572	4112
Vitamin D_3_, IU	761	795
Vitamin E, IU	30	31
Vitamin K, μg	321	362
Thiamin, μg	1322	1588
Riboflavin, μg	2608	2780
Niacin, μg	13,366	11,132
Vitamin B_6_, μg	1210	1414
Folic acid, μg	211	237
Vitamin B_12_, μg	6	7
Pantothenic acid, μg	9216	8170
Biotin, μg	74	74
Choline, mg	352	394
Polydextrose, g	1.8	1.9
Galactooligosaccharide, g	2.1	1.7
Arachidonic acid, mg	318	288
Docosahexaenoic acid, mg	155	141

**Table 2:** Effects of dietary sialyllactose supplementation on the growth performance of pigs over the duration of the feeding study ^1^.

	Diet ^2^		Pooled	
Measure ^3^	Control	Sialyllactose	SEM	*p*-Value ^4^
ADG, g/day	311	306	14	0.69
ADMI, g milk/day	1220	1347	62	0.11
ADMI, g solids/day	244	269	12	0.11
G:F, g BW:kg milk	255	234	11	0.16

^1^ Abbreviations: SEM, standard error of the mean; BW, body weight; kg, kilogram; ADG, average daily body weight gain; ADMI, average daily milk intake; G:F, gain-to-feed ratio (i.e., feed efficiency); ^2^ *n* = 17 per diet; ^3^ Calculations reflect a milk reconstitution rate of 20% solids; ^4^ *p*-values derived from mixed model ANOVA.

**Table 3:** Ability of pigs to display a recognition index score above 0.50 as a measure of recognition memory in the NOR test trial ^1^.

Diet	*n*	Mean	SEM	*p*-Value ^2^
Control	15	0.65	0.046	<0.01
Sialyllactose	12	0.66	0.047	<0.01

^1^ Abbreviations: NOR, novel object recognition; SEM, standard error of the mean; ^2^ *p*-Value derived from one-tailed *t*-test for a recognition index above 0.50.

**Table 4:** Effect of dietary sialyllactose supplementation on exploratory behavior during the test trial of the NOR task ^1^.

	Diet					
	Control		Sialyllactose		Pooled	
Measure	*n*	Mean	*n*	Mean	SEM	*p*-Value ^2^
Recognition index	15	0.66	12	0.65	0.05	0.94
Novel object visit time, s	15	56.63	12	42.05	7.77	0.18
Number of novel object visits	15	8.33	12	6.78	1.07	0.25
Mean novel object visit time, s	15	6.19	11	6.41	1.12	0.88
Latency to first novel object visit, s	15	25.46	12	25.32	9.31	0.99
Habituation towards the novel object, s/min	15	−1.60	12	−0.69	1.25	0.59
Sample object visit time, s	14	28.27	12	22.45	6.45	0.50
Number of sample object visits	15	4.77	12	4.62	0.56	0.84
Mean sample object visit time, s	15	6.18	12	5.51	1.34	0.71
Latency to first sample object visit, s	14	24.08	12	14.25	7.15	0.31
Habituation towards the sample object, s/min	14	−2.68	12	−1.78	0.73	0.35
Total object visit time, s	15	82.52	12	70.35	13.51	0.47
Mean object visit time, s	15	7.10	12	6.11	1.22	0.55
Number of object visits	15	13.13	12	11.42	1.34	0.31
Latency to first object visit, s	15	9.48	12	13.50	4.97	0.55
Habituation towards both objects, s/min	15	−4.32	12	−1.76	1.39	0.19
Total distance moved, m	15	2.43	11	2.11	0.19	0.11
Time spent in the center of the arena, %	15	58.76	12	56.73	6.95	0.80

^1^ Abbreviations: NOR, novel object recognition; SEM, standard error of the mean; ^2^ *p*-Values derived from mixed model ANOVA.

**Table 5:** Effect of dietary sialyllactose supplementation on diurnal activity of pigs ^1^.

	Diet ^2^											
	Control				Sialyllactose							
	Day		Night		Day		Night		Pooled	*p*-Value ^3^		
Measure	*n*	Mean	*n*	Mean	*n*	Mean	*n*	Mean	SEM	Diet	Cycle	Interaction
Total activity count	65	1.6 × 10^5^	70	6.9 × 10^4^	67	1.7 × 10^5^	72	6.9 × 10^4^	7.0 × 10^3^	0.64	<0.01	0.56
Time asleep, %	65	67.47	70	84.90	71	67.43	72	85.63	0.97	0.61	<0.01	0.58

^1^ Abbreviations: SEM, standard error of the mean; %, percent; ^2^ Data from 12 pigs per diet over a six-day period; ^3^ *p*-Value derived from repeated measures mixed model ANOVA.
